# Impact of First Meal Size during Prolonged Sitting on Postprandial Glycaemia in Individuals with Prediabetes: A Randomised, Crossover Study

**DOI:** 10.3390/nu10060733

**Published:** 2018-06-06

**Authors:** Evelyn B. Parr, Brooke L. Devlin, Samuel K. Pinto, David W. Dunstan, John A. Hawley

**Affiliations:** 1 Exercise and Nutrition Research Program, Mary MacKillop Institute for Health Research, Australian Catholic University, Fitzroy, VIC 3065, Australia; brooke.devlin@acu.edu.au (B.L.D.); samuel.pinto@myacu.edu.au (S.K.P.); david.dunstan@baker.edu.au (D.W.D.); john.hawley@acu.edu.au (J.A.H.); 2 Baker Heart and Diabetes Institute, Melbourne, VIC 3004, Australia

**Keywords:** glyacemic control, breakfast, obesity, sedentary behavior, insulin, energy distribution

## Abstract

We compared the impact of a high versus low energy intake first meal on glucose and insulin responses during prolonged sitting in individuals with prediabetes. Thirteen adults with overweight/obesity and prediabetes (mean ± SD age: 60 ± 6 years, BMI: 33 ± 4 kg/m^2^; 2 h OGTT: 8.9 ± 1.1 mmol/L) completed two randomised trials: 10 h uninterrupted sitting, incorporating three meals with matching macronutrient compositions but different energy distributions: High-Energy Breakfast (HE-BF; breakfast: 50%, lunch: 30%, dinner: 20% energy intake), Low-Energy Breakfast (LE-BF: 20%/30%/50% energy intake). Venous blood was sampled from 08:00–18:00 h for determination of plasma glucose and insulin concentrations, with 24 h continuous glucose monitoring (CGM). Total glucose area under the curve (AUC; +5.7 mmol/L/h, *p* = 0.019) and mean plasma glucose concentrations (+0.5 mmol/L, *p* = 0.014) were greater after HE-BF compared to LE-BF. In the HE-BF condition, compared to LE-BF, there was a greater incremental area under the curve (iAUC) for plasma glucose post-breakfast (+44 ± 59%, *p* = 0.007), but lower iAUC post-lunch (−55 ± 36%, *p* < 0.001). Total insulin AUC was greater (+480 mIU/mL/h, *p* < 0.01) after HE-BF compared to LE-BF. Twenty-four-hour (24 h) CGM revealed no differences in mean glucose and total AUC between conditions. Compared to a low-energy first meal, a high-energy first meal elicited exaggerated plasma insulin and glucose responses until lunch but had little effect on 24 h glycaemia. During periods of prolonged sitting, adults with prediabetes may have more beneficial postprandial insulin responses to a low-energy first meal.

## 1. Introduction

The continued rise in the prevalence of obesity and the rate of diagnosis of individuals with type 2 diabetes (T2D) imposes a substantial burden on healthcare systems. As such, adults with prediabetes, who spend a large proportion of their day in a state of postprandial hyperglycaemia [1], have become a key target group for preventive lifestyle interventions in an effort to prevent T2D [2] and associated cardiovascular complications [3]. The magnitude of postprandial glucose excursions across the day and night have become a major clinical focus in treatment strategies for individuals with prediabetes [4]. Understanding the extent to which behavioural factors, such as meal size and prolonged periods of inactivity, influence the postprandial state is essential for future management of adults with prediabetes.

In recent decades, substantial changes in the modern environment have been evident, specifically increases in the time spent in sedentary behaviour (sitting). Sedentary behaviour is associated with increased risk of T2D even after accounting for habitual physical activity levels [5,6]. Both observational and experimental studies demonstrate that prolonged sitting is detrimental to postprandial glucose levels [7], where the greatest exaggeration in hyperglycaemia with prolonged sitting occurs in the period after the first meal [8,9]. Therefore, optimising the daily distribution of energy may be a practical strategy for modifying postprandial hyperglycaemic responses to meals.

Several studies have shown reductions in body mass, waist circumference and daily hyperglycaemia following 7 days to 12 weeks of consuming a large breakfast (50% energy intake (EI)) meal compared to an energy-matched large dinner meal during energy restriction, in both individuals with metabolic syndrome [10] and T2D [11,12]. Despite the reduction in daily hyperglycaemia, exaggerated postprandial hyperglycaemia at breakfast was observed when a large breakfast was consumed compared to a smaller, typical breakfast-sized meal (~20% EI). However, a shortcoming when interpreting the results from these interventions is that they have used a protocol whereby the energy content of the meal and the macronutrient composition have been simultaneously altered. Specifically, a higher carbohydrate (CHO) meal in the morning (45% CHO, 25% protein), rather than at the end of the day, has been shown to improve total daily area under the curve (AUC) compared to a smaller breakfast first meal higher in protein (10% CHO, 65% protein) [10,12]. Subsequently, the large morning meal was balanced by a smaller, high-protein meal in the evening. As insulin sensitivity is greatest upon waking compared to the end of the day [13], the larger CHO meal consumed at the beginning of the day is ingested when insulin sensitivity (i.e., beta-cell function) is highest. Furthermore, the composition of the first meal has been shown to influence substrate oxidation for the following 24 h [14], while skipping breakfast altogether is associated with greater risk of developing T2D [15]. To date, no meal-size investigation has been performed in individuals with prediabetes, who exhibit high peak glucose values in the morning irrespective of meal size [16,17]. Therefore, it is important that the macronutrient composition of each meal investigated is consistent so that the energy intake at each meal can be attributed to the observed outcomes.

To date, no study has determined the relative impact of energy intake or macronutrient composition on daily glucose and insulin levels in adults with prediabetes. Furthermore, the impact of meal size during prolonged periods of sitting in adults with prediabetes has not been examined. Accordingly, the aim of the present investigation was to determine how manipulations to the distribution of energy throughout the day, with meals of the same macronutrient composition, affect blood glucose and insulin concentrations during prolonged sitting in men and women with overweight/obesity and prediabetes. We hypothesised that a high-energy first meal would result in a less favorable postprandial glucose and insulin profile throughout a day of prolonged sitting than a low-energy first meal.

## 2. Materials and Methods

### 2.1. Participants

Men and women (aged 40–70 years) with overweight/obesity (body mass index (BMI) ≥ 25 but <45 kg/m^2^), sedentary (i.e., watching at least 3 h television per day) and inactive (i.e., not meeting physical activity guidelines of ≥150 min/week of moderate-intensity exercise for >3 months) lifestyles, who presented with impaired fasting glucose (IFG; ≥6.0 mmol/L but <7.1 mmol/L) and/or impaired glucose tolerance (IGT) after 2 h of a 75 g oral glucose tolerance test (OGTT; ≥7.8 mmol/L but <11.0 mmol/L) [18] were recruited from local community advertisements. Exclusion criteria were: diagnosis of T2D; pregnancy; current smoker; employment in a non-sedentary occupation; previous bariatric surgery; and medications known to impact on measurements of blood glucose. Following initial phone screening, potentially eligible participants attended laboratory screening, which included written consent, fasting and 2 h OGTT blood sampling (*n* = 51). A separate subset of individuals completed pre-laboratory HbA1c testing at a local pathology clinic (*n* = 10), where ≥6.0% (42 mmol/mol) HbA1c indicated potential eligibility and subsequently they attended confirmatory laboratory OGTT testing (*n* = 1).

### 2.2. Study Design

This randomised crossover study was undertaken at the Australian Catholic University (ACU) between February–September 2016 (Australian New Zealand Clinical Trial Registry number ACTRN12615001216505) after approval from the ACU Human Research Ethics Committee (2015-209H). Eligible participants attended the laboratory on seven separate occasions: Once for pre-trial body composition (dual-energy X-ray absorptiometry (DXA) and resting energy expenditure measurement (REE); and three visits per trial: (i) pre-trial glucose monitor insertion; (ii) trial day (07:30 to 18:15 h); and (iii) post-trial visit (08:00 h next day). Two trial conditions were completed by each participant in a randomised order, separated by at least 10-days washout (no monitoring of diet or physical activity). Trial condition order was randomly assigned by a third party using computer-generated random numbers and sealed opaque envelopes (block randomised, *n* = 4) and revealed to study personnel on the day prior to the first trial.

### 2.3. Preliminary Measures

An initial screening visit confirmed prediabetes status by HbA1c testing and/or a 2 h OGTT (75 g glucose solution (PoC Diagnostics, North Rocks, NSW, Australia), analysed on the YSI 2900 (YSI Life Sciences, Yellow Springs, OH, USA)). Eligible participants attended pre-trial fasted measurements of body composition (DXA; GE Lunar iDXA Pro, enCORE software Version 16) and REE using a metabolic cart (TrueOneRMR, Parvo Medics, Sandy, UT, USA), calibrated for expired gas (15.99% O_2_, 1.00% CO_2_) and flow rate to determine resting energy expenditure (REE; kJ/d) from the average of the final 20 of 45 min of data collected.

### 2.4. Study Protocol and Trial Conditions

**Figure 1:**
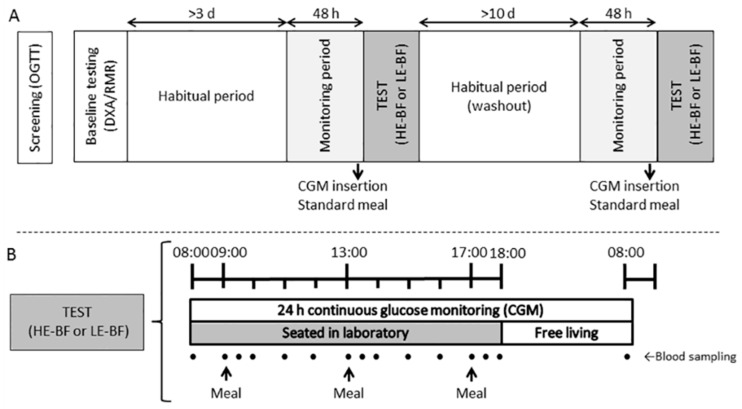
Study protocol: (**A**) overview of the entire study; (**B**) Overview of the trial days. Eligible participants (*n* = 13) with prediabetes completed two trial conditions in a randomised order separated by >10 days. Blood was collected hourly and 30 min post each meal. Meals were provided at 09:00, 13:00 and 17:00 h in different energy distribution relative to the condition (HE-BF: 20/30/50% EI; LE-HF: 50/30/20% EI) with the same macronutrient composition in each meal (50% carbohydrate, 20% protein and 30% fat).

Figure 1 shows the study protocol. Participants wore activity monitors (SenseWear Armband (SWA), Bodymedia, Pittsburgh, PA, USA; activPAL3 tri-axial accelerometer, PAL-technologies Ltd., Glasgow, Scotland) for 48 h prior to and throughout each trial. Participants were asked to refrain from moderate-vigorous physical activities (MVPA) for 48 h and caffeine and alcohol consumption for 24 h prior to each experimental condition (verified using activity monitors and food diaries). Participants on medications were instructed to take and record medications as usual on testing days.

Participants attended the laboratory the day before each trial to have a continuous glucose monitor (CGM; iPro2 with Enlite sensor; Medtronic, Northridge, CA, USA) inserted into the subcutaneous tissue of their lower back, adhered with waterproof dressings; and were provided with a hand-held glucometer (Accu-Chek Performa II, Roche Diagnostics Ltd., Rotkreuz, Switzerland) for CGM calibration 1 h post insertion, pre-dinner and pre-sleep; and a standardised meal (33% daily EI; 50% CHO, 30% fat, 20% protein) to consume between 18:00 and 20:00 h. On the trial day, the CGM was calibrated with finger stick measures pre-breakfast, -lunch, -dinner and -sleep, as well as fasting the following morning.

On the morning of each experimental condition, participants arrived at the laboratory at ~07:30 h and an indwelling cannula (22G; Terumo, Tokyo, Japan) was inserted into an antecubital forearm vein of each participant. A blood sample (12 mL; EDTA) was taken and the cannula was regularly flushed with saline. Participants were seated on a sofa chair by 08:00 h and instructed to remain seated, with toilet breaks permitted when required (matched in the subsequent trial), throughout the trial period until 18:00 h when the cannula was removed, and participants returned home. At ~07:45 h the next morning, participants returned in a fasted state for a final blood sample and removal of monitors. 

During each trial day, serial blood samples (12 mL, EDTA) were collected hourly and 30 min post meals. Whole blood (1 mL) was transferred into a separate tube containing a DPP-IV inhibitor (Merck Millipore, Darmstadt, Germany) additive for total glucagon-like peptide 1 (tGLP-1) stability, prior to centrifugation. The EDTA and tGLP-1 tubes were centrifuged at 1800 *g*, 10 min at 4 °C. Plasma was immediately aliquoted and stored at −80 °C for later analysis. 

Trial conditions were based on a high-energy breakfast (HE-BF) with 50% EI at breakfast and 20% EI at dinner or a low-energy breakfast (LE-BF) with 20% EI at breakfast and 50% EI at dinner. Both conditions had the same lunch (30% EI). Participants consumed meals within 20 min and water ad libitum during the first trial condition (matched in the subsequent trial). After the evening meal, participants were asked not to consume any other energy-containing foods or beverages (except water) until they returned to the laboratory the following morning.

#### Meal Composition

Meals were provided to participants throughout each trial day with breakfast at 09:00 h, lunch at 13:00 h and dinner at 17:00 h. Total EI was individualised based on REE x 1.5 physical activity factor. Each meal composition was 50% energy from CHO, 30% energy from fat and 20% energy from protein. Foods selected for each meal (see Supplementary information, Table S1) were chosen to meet the required composition and have a similar glycaemic index. Fibre content differed due to meal size differences.

### 2.5. Biochemical Analysis

Plasma glucose concentration was measured using a hexokinase method at a commercial laboratory (Melbourne Pathology, Collingwood, Victoria, Australia) with a CV < 1.9%. Fasting triglycerides were analysed using a Cobas b 101 instrument (Roche Diagnostics Ltd., Basel, Switzerland). Plasma insulin, connecting peptide (C-peptide) and tGLP-1 concentrations were measured using a Luminex Analyzer (MAGPIX^®^; Human Metabolic Hormone Magnetic Bead Panel, EMD Millipore, Burlington, MA, USA) following manufacturer’s instructions with intra-assay CV’s of 9.8%, 6.8% and 11.4%, respectively.

### 2.6. Data Analysis

Incremental area under the curve (iAUC) was used to assess the 4-h post-meal periods following breakfast and lunch for venous plasma glucose for each condition (secondary outcome). The iAUC was calculated using the trapezoidal rule to measure net AUC, with the initial measure prior to meal commencement as baseline. Total AUC for plasma glucose, insulin, c-peptide, and tGLP-1 total from 08:00 to 17:00 h were calculated using the trapezoidal rule with a zero baseline. For CGM data, iAUC was calculated using the trapezoidal rule to measure the net AUC, with the 15 min period prior to each meal commencement as baseline. Total AUC was calculated from CGM data from 08:00 to 18:00 h to compare with venous measurements, as well as from 08:00 for 24 h total AUC. Mean 24 h glucose concentration, the standard deviation (SD_glucose_) over 24 h, and measures of glycaemic variability (mean amplitude of glycaemic excursions (MAGE) [19], continuous overall net glycaemic action for 1 h (CONGA1) [20]) were analysed from CGM data using EasyGV [21]. Homeostatic model assessment (HOMA of beta-cell function (HOMA2-%beta), insulin sensitivity (HOMA2-%S) and insulin resistance (HOMA2-IR)) were calculated, using insulin concentrations [22].

Time spent sitting/lying, standing and stepping were estimated from activPAL3 data for the waking periods (ascertained from self-reported sleep and waking times) of the habitual (pre-trial) days and trial days using SAS 9.4 (SAS Institute, Cary, NC, USA), as described [8]. SWA generated estimates of daily EE were measured through the recorded data based on the SWA proprietary algorithm, as previously validated [23], using SenseWear Professional 7.0 software (Bodymedia Inc., Pittsburgh, PA, USA).

#### 2.6.1. Sample Size Calculations

Sample size calculations for the primary outcome of venous glucose concentrations were based on mean daily glucose (from venous collections). Based on previous behavior and nutrition interventions [10,24], it was estimated that a sample size of 22 would be needed to detect a repeated measure between a treatment difference of 0.9 ± 1.04 mmol/L with a minimum power of 80% and α = 0.05 (two-tailed test; G*Power 3.1.2 software). The sample size in the current study is less than our original power calculations predicted to detect differences between treatments, but is similar to other interventions that have investigated glycaemic responses in individuals with IGT [16,17,25]. The trial ended prior to reaching our target sample size as a result of practical difficulties (each trial required two full days in the laboratory) and the lower than expected potential participants with IFG/IGT (21% of eligible participants). As a consequence, the study is likely to have been underpowered to detect significant differences. Therefore, the following analyses should be considered exploratory. 

#### 2.6.2. Statistical Analysis

Statistical analyses were performed using SPSS (Version 22, SPSS Inc., Chicago, IL, USA). Linear mixed models (LMM), using a scaled identity covariance structure, were used to analyse baseline data and changes across time, using intervention allocation as the grouping variable and time spent in MVPA (from SWA) in the 48 h pre-trial as a covariate. For the primary outcome of venous glucose concentrations overtime, significance was set at *p* < 0.05 and post-hoc comparisons between the groups were conducted when significant within the LMM based on the least significant squares (LSD) test. While numerous analyses were conducted, no post-hoc corrections were made to account for Type 1 error due to the exploratory nature of the analyses of secondary outcomes; therefore, only main effects are reported. Residuals from the LMM were plotted to assess and confirm normally distributed data. All data are presented as mean ± SD, with mean differences and 95% confidence intervals (CI) where appropriate.

## 3. Results

### 3.1. Participant Characteristics

**Figure 2:**
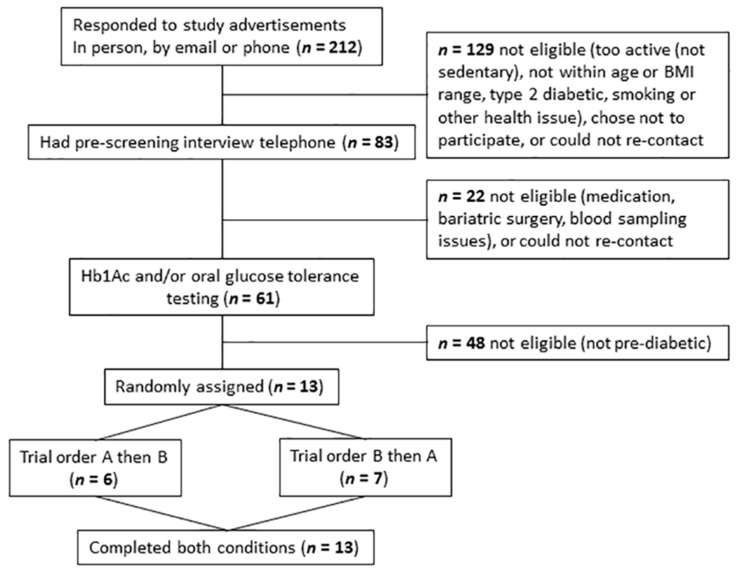
Consolidated Standards of Reporting Trials (CONSORT) flow diagram of participant recruitment. For a full CONSORT statement see Supplementary File S1.

Of the 61 participants screened, 13 eligible participants (7 females, 6 males; mean ± SD, age: 60 ± 6 years; BMI: 33 ± 4 kg/m^2^; body mass (BM): 91.2 ± 11.9 kg; BF%: 43 ± 7%; fasting glucose: 5.6 ± 0.8 mmol/L; 2 h OGTT glucose: 8.9 ± 1.1 mmol/L) were randomised and completed both conditions (Figure 2). Twelve participants were classified as having IGT, with one participant classified as having IFG, with *n* = 8 taking medication (for hypertension (*n* = 4), hypercholesterolemia (*n* = 3), thyroid activity (*n* = 2) and depression (*n* = 2)). No differences were observed between conditions at baseline, for anthropometric, biochemical, physical activity patterns or dietary intake (Table 1).

### 3.2. Interventions

Participants consumed 11,528 ± 1882 kJ in each condition across a trial day. Habitual dietary intake patterns were distributed similarly to the LE-BF condition, whereby participants consumed 19–21% EI at breakfast, 31–38% EI at lunch and 42–48% EI at dinner. Three-day food record analysis revealed similar habitual daily macronutrient distributions to the trial meals (CHO (including fibre): ~48% EI; Fat: ~31% EI; Protein: ~19% EI).

Physical activity levels were low prior to both conditions, corresponding with low levels of habitual EE in the 48 h pre-trial period (Table 1). On the day of trial, total daily EE and activity patterns were similar between conditions (Table 2). The trial period differed to the habitual pre-trial period whereby the daily EE was reduced (*p* < 0.001; Table 1 and Table 2), the proportion of time standing and moving was reduced and the proportion of time sitting increased (all *p* < 0.001), including the accumulated amount of time spent sitting for >30 min blocks (*p* < 0.001; Table 2).

### 3.3. Glycaemic Control

**Figure 3:**
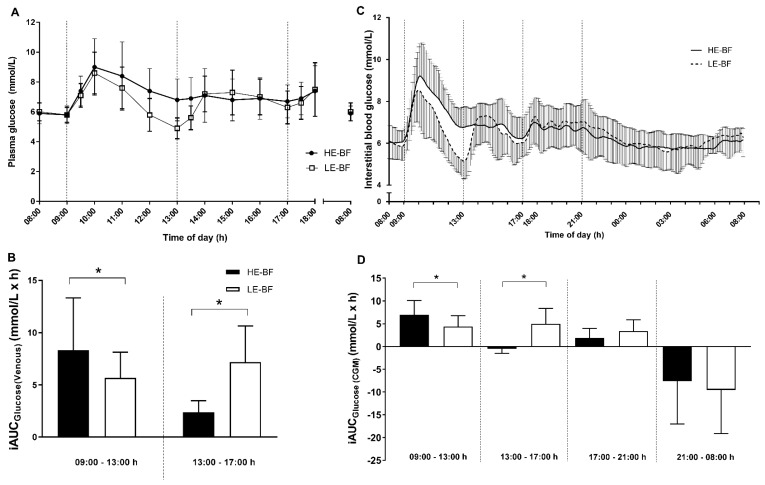
Venous glucose concentrations (**A**) from 1 h pre-breakfast (08:00) until 1 h post-dinner (18:00), for participants with prediabetes (*n* = 13) throughout trial conditions (high-energy breakfast (HE-BF; black bars and symbols) and low-energy breakfast (LE-BF; white bars and symbols)), and venous incremental area under the curve (iAUC) (**B**) for 4 h meal periods (09:00–13:00 h; 13:00–17:00 h); Interstitial (CGMS) glucose (**C**) values from one hour pre-breakfast (08:00) until 08:00 the following morning, and CGM iAUC (**D**) for 4 h meal periods (09:00–13:00 h; 13:00–17:00 h; 17:00–21:00 h) and overnight (21:00–08:00 h). Meals were ingested at 09:00, 13:00 and 17:00 h on both condition days. Vertical lines represent the 4 h iAUC analysis periods. Data are mean ± SD. From LMM analyses, significantly different * between groups (*p* < 0.05).

Mean plasma glucose from venous samples was higher in HE-BF (7.1 ± 1.1 mmol/L) compared to the LE-BF condition (6.7 ± 0.8 mmol/L; +0.5 mmol/L, 95% CI: 0.1–0.9 mmol/L; *p* = 0.014; Figure 3A). Accordingly, plasma glucose AUC_total_ was greater in the HE-BF (71.5 ± 12.0 mmol/h/L) compared to the LE-BF condition (66.8 ± 7.8 mmol/h/L; +5.7 mmol/h/L, 95% CI: 1.2–10.1 mmol/h/L; *p* = 0.019). The postprandial glycaemic response, measured by iAUC in the 4 h post-breakfast period was 44 ± 59% greater in the HE-BF condition compared to LE-BF (+2.9 mmol/h/L, 95% CI: 0.4–5.4 mmol/h/L; *p* = 0.03; Figure 3B). There were no differences between conditions in peak glucose post-breakfast (HE-BF: 9.0 ± 1.9 mmol/L; LE-BF: 8.6 ± 1.4 mmol/L; +0.4 mmol/L, 95% CI: −0.3–1.1 mmol/L). However, in the 4 h post-lunch meal period a 55 ± 36% greater iAUC was observed in the LE-BF condition (+4.8 mmol/h/L, 95% CI: 2.6–7.0 mmol/h/L; *p* = 0.02; Figure 3B), due to a decrease in plasma glucose below baseline levels in the LE-BF condition prior to the lunch meal (Figure 3A).

Interstitial CGM results were similar to venous glucose for total AUC_8-6_ (i.e., a comparative time measurement, Table 3), and for the iAUCs for the post-breakfast (HE-BF: +105 ± 133% greater; +2.9 mmol/h/L, 95% CI: 1.0–5.0 mmol/h/L; *p* = 0.007) and -lunch (LE-BF: +109 ± 33% greater; +5.3 mmol/h/L, 95% CI: 3.4–7.1 mmol/h/L; *p* < 0.001;) periods (Figure 3D). The 4 h post-dinner iAUC was similar between conditions (+1.5 mmol/h/L, 95% CI: −0.2–3.2 mmol/h/L; *p* = 0.08; Figure 3D). Despite differences in CGM iAUC, total AUC and mean glucose from CGM over 24 h were not different between conditions (Figure 3C and Table 3).

A similar pattern for glucose response to meals was observed in both venous and CGM glucose measures (Figure 3A,C). Plasma glucose concentrations were higher from 30 min to 2 h post-breakfast in the HE-BF condition but decreased below baseline just before lunch in the LE-BF condition. No differences between conditions were observed the morning following each trial for venous glucose concentrations (Figure 3A) or in CGM iAUC overnight (21:00–08:00 h) (Figure 3D). Despite differences in CGM iAUC for the first two meal periods, overall measures of glycaemic variability were not different between conditions (SD, MAGE and CONGA1; Table 3).

### 3.4. Insulin, C-Peptide and tGLP-1

**Figure 4:**
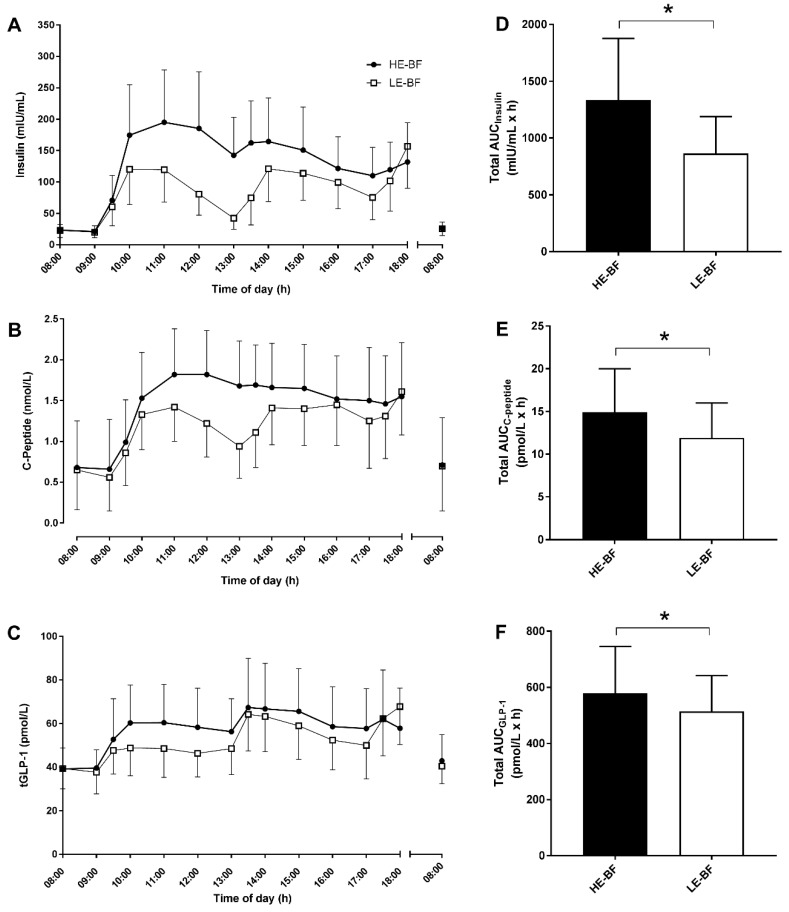
Plasma hormone concentrations of Insulin (**A**), C-peptide (**B**), and tGLP-1 (**C**), from participants with prediabetes (*n* = 13) throughout (08:00–18:00 h) trial conditions (high-energy breakfast (HE-BF; black bars and symbols) and low-energy breakfast (LE-BF; white bars and symbols)) and the morning after (08:00 h); and the associated total AUC (08:00–17:00 h) for insulin (**D**), C-peptide (**E**) and tGLP-1 (**F**). Data are mean ± SD. From LMM analyses, * significantly different (*p* < 0.01) between conditions.

A main effect of time was observed as plasma insulin concentrations increased from 30 min post-breakfast in both conditions (*p* < 0.001) to a larger magnitude in the HE-BF condition (time × group: *p* < 0.001; Figure 4A). Insulin was higher in the HE-BF than the LE-BF condition until 2 h post-lunch (*p* < 0.03) and was elevated above baseline from 1 h post meal for the entire day (*p* < 0.001). Insulin concentrations in the LE-BF condition returned to concentrations similar to baseline at pre-lunch (13:00 h, *p* = 0.39), but were elevated above baseline for all other time points (*p* < 0.04). 

C-peptide and tGLP-1 concentrations were elevated above baseline from 30 min post-breakfast until 18:00 h (both main effect, time: *p* < 0.001; Figure 4B,C). From 2 h post-breakfast to 30 min post-lunch, C-peptide concentrations were lower in LE-BF compared to HE-BF (*p* < 0.03). Greater concentrations of tGLP-1 were observed 1 h post-breakfast to pre-lunch in the HE-BF condition (main effect: group: *p* < 0.001). Consequentially, total AUC for insulin, C-peptide and tGLP-1 measured between 08:00 and 17:00 h was greater in the HE-BF compared to the LE-BF condition (*p* < 0.01, Figure 4D–F). No differences were observed between conditions in fasting insulin, C-peptide, or tGLP-1 concentrations the morning following each trial.

## 4. Discussion

This is the first study to investigate acute postprandial responses to a high- versus low-energy first meal of the same macronutrient composition during a day of prolonged uninterrupted sitting in individuals with prediabetes. The study design is novel since energy intake at each meal was experimentally manipulated but importantly, and in contrast to previous investigations, the macronutrient composition of each meal was matched. We observed that the first feeding occasion was the “priming meal” for subsequent glucose responses, independent of energy content, but by early afternoon perturbations in glucose and insulin were similar in response to both feeding patterns. No differences were observed in mean 24 h glucose or AUC from CGM analyses, but differences in blood glucose patterns throughout the morning and following lunch were evident.

Over the long term glycaemic control, and in particular glycaemic variability (i.e., peaks and nadirs), is clinically relevant in the development of complications (such as impaired endothelial function and damage due to oxidative stress) associated with type 2 diabetes [26,27]. In our cohort of individuals with prediabetes, glycaemic variability measures did not differ between conditions. Furthermore, we found similar peak venous plasma glucose concentration after the first meal despite the vastly different energy content (50% vs. 20% of total EI). Hence, the observed benefits to glycaemic control from a large first meal that has previously been demonstrated are most likely due to the disparity in meal *composition* (i.e., CHO content) of the meals [10,12]. In the context of Western breakfast meal composition as used in the current intervention, the effects of skewing energy intake towards a large first meal did not acutely improve daily glycaemic control. Whether chronic adoption of a large-CHO-based first meal [12] improves daily glycaemic control for individuals with prediabetes requires further investigation. Previous investigations have fed a greater proportion of daily CHO intake into the first meal [10,12], rather than a more even distribution of energy across lunch and evening meals, to show improved glycaemic control. Accordingly, the macronutrient composition of the meal in conjunction with the energy content is an essential consideration when manipulating meal size and timing to improve postprandial and daily rhythms of glucose in individuals with prediabetes.

In the current investigation, the relatively small iAUC glucose response post-dinner meal in both conditions, compared to breakfast, highlights the importance of the first meal for control of daily hyperglycaemia in individuals with prediabetes. These findings support previous reports of exaggerated peak blood glucose responses in individuals with prediabetes after the first meal when meal size, independent of composition, is the same [17] or smaller [16]. Despite the high-energy first meal eliciting the largest perturbations in postprandial blood glucose concentrations, such differences did not persist throughout the 24 h monitoring period. Consequently, to counteract the large and prolonged rise in blood glucose observed in the high-energy compared to the low-energy first meal condition, an exaggerated morning insulin response was observed.

Elevated insulin concentrations have been observed in individuals with T2D following consumption of a large breakfast [12], but not in response to a chronic large breakfast meal pattern in women with overweight or obesity [10]. The return to baseline in plasma insulin concentrations before lunch in the low-energy first meal condition suggests the smaller first meal allows insulin-resistant individuals to process the glucose load without excess insulin secretion, thereby lowering the total AUC insulin. Given the hyperinsulinemic and hyperglycaemic consequences of a high-energy first meal observed in individuals with prediabetes, it could be speculated that such an energy distribution pattern in the long term may lead to the development of defective beta-cells, reducing insulin secretion and impairing glucose tolerance [28].

Plasma C-peptide concentrations tracked the insulin responses and provided an indirect measure of the insulin-secretory activity of the beta-cells [29]. C-peptide concentrations did not return to baseline levels throughout either condition, most likely due to the slow clearance from the circulation [30]. Total GLP-1 was elevated 1 h post-first meal in both conditions but to a greater extent with a high-energy first meal condition. The tGLP-1 responses to both conditions showed a lack of oscillations between meals that have been observed in healthy individuals [30]. Unlike insulin and C-peptide, differences in tGLP-1 were only observed between conditions until the second meal and the elevation of tGLP-1 in both conditions after the second meal supports the second meal phenomenon of incretin hormones playing an important role in attempting to reduce postprandial hyperglycaemic responses [12]. While the mechanisms underlying the second meal phenomenon are unclear, for individuals with T2D the second meal is potentiated by the insulin secretion in response to the first meal [31], as presently observed for responses to both large and small first-meals in individuals with prediabetes.

We chose to investigate individuals with prediabetes since the transition from prediabetes to T2D has been shown to be delayed or prevented through lifestyle interventions [32,33,34]. Furthermore, the circadian responses of glucose and insulin of healthy, normal weight participants, where glucose tolerance and insulin sensitivity are reduced in the evening [13,35], are different to those in individuals with T2D [36]. The use of CGM allowed for the measurement of total AUC glucose across a 24 h period, rather than simply adding the post meal AUCs. It also provides insights into the changes in interstitial glucose between meals and lack of change in interstitial blood glucose throughout the night. While there were small or no differences between the conditions in these measures, there were inter-individual differences within the cohort highlighting the variable nature of IGT/IFG.

We maintained strict control of activity levels throughout the day of the experimental intervention to replicate the modern environment where obesity and T2D diagnosis are often synonymous with decreased physical activity. As the post-first meal period is important for daily glucose control for individuals with prediabetes [16,17], incorporation of specific post-breakfast physical activity could be a prudent approach to optimise postprandial hyperglycaemic responses to any first meal, regardless of size. Indeed, there is growing evidence supporting activity after mealtimes reducing postprandial glucose and 24 h glucose concentrations in individuals with T2D [25,37,38].

A limitation of the present study is the lack of venous sampling after 1800 h and consequently the inability to characterise the postprandial insulin, C-peptide or GLP-1 responses after the dinner meal. Furthermore, the timing of meals was similar to a time-restricted feeding protocol [39] which may have exacerbated the postprandial hyperglycaemic response to the lunch and dinner meals. While the randomised within-subjects study design a powerful we acknowledge that the sample size was smaller than originally intended. Despite multiple secondary outcome analyses, we did not correct for multiple comparisons given the exploratory nature of the analyses and the importance of identifying variables of interest for further study. Consequently, there is a risk of a Type 1 error and therefore results should be interpreted with caution. These considerations highlight the need for a larger, longer-term intervention, where meal size is the modifiable factor and each meal composition is controlled. However, food availability/choices at mealtimes often dictate the composition so there is also a need to investigate meal size with the consideration of typical meal compositions (i.e., higher carbohydrate intakes at breakfast and higher protein at dinner).

## 5. Conclusions

Our results support a role for first meal energy intake to influence postprandial glucose and insulin responses in adults with prediabetes, independent of macronutrient composition. In contrast to the results of studies of meal energy distribution, the clinical advantage of a high-energy first meal to improve daily blood glucose regulation was not evident in this investigation in individuals with prediabetes. We conclude that for adults with prediabetes who experience prolonged sedentary periods, particularly in the hours leading to lunch, a low-energy first meal may be desirable for postprandial glucose and insulin regulation, although 24 h glycaemic control was not impaired or improved by a high-energy first meal of the same composition. 

## Figures and Tables

**Table 1:** Anthropometric, biochemical, physical activity and dietary information from participants with prediabetes (*n* = 13) prior to each trial condition.

	Condition	
	HE-BF	LE-BF
Body mass (trial day; kg) ^a^	91.6 ± 13.8	91.3 ± 13.8
Body composition ^b^		
Body mass (kg)	91.5 ± 12.4	
Fat mass (kg)	38.2 ± 7.1	
Lean mass (kg)	50.7 ± 10.4	
Visceral adipose tissue (kg)	2.1 ± 0.7	
Fasting concentrations ^c^		
Plasma glucose (mmol/L)	5.52 ± 0.76	5.22 ± 0.63
Plasma insulin (mIU/mL)	23.6 ± 13.4	21.7 ± 10.8
Plasma C-peptide (nmol/L)	0.69 ± 0.66	0.64 ± 0.49
Plasma tGLP-1 (pmol/L)	40.6 ± 9.6	39.7 ± 10.1
Triglycerides (mmol/L)	1.47 ± 0.51	1.69 ± 0.61
Total Cholesterol (mmol/L)	4.79 ± 1.42	5.06 ± 1.40
HDL cholesterol (mmol/L)	1.02 ± 0.28	1.03 ± 0.23
LDL cholesterol (mmol/L)	3.11 ± 1.27	3.24 ± 1.21
HOMA2-%beta	165 ± 80	183 ± 75
HOMA2-%S	41 ± 15	44 ± 19
HOMA2-IR	2.84 ± 1.21	2.74 ± 1.24
Physical activity time (min/day) ^d^		
Light-intensity	156 ± 52	149 ± 42
Moderate-intensity	49 ± 38	48 ± 24
Vigorous-intensity	0 ± 0	0 ± 0
Proportion of waking hours spent sedentary (%) ^e^	65 ± 12	67 ± 12
Estimated daily energy expenditure (kJ/day) ^d^	10,296 ± 2275	9772 ± 1461
Diet		
Total energy intake (kJ/day)	7665 ± 2651	7519 ± 3128
Total carbohydrate (% of energy intake)	46.0 ± 7.0	46.0 ± 8.6
Total fat (% of energy intake)	31.6 ± 8.0	30.1 ± 5.6
Total protein (% of energy intake)	18.6 ± 5.3	19.3 ± 5.2
Resting energy expenditure (kJ/day) ^f^	7311 ± 1221	

Data are mean ± SD. Key: C-peptide, connecting peptide; tGLP-1, total glucagon like peptide 1; HE-BF, high-energy breakfast condition; HOMA, homeostatic model assessment; IR, insulin resistance; LE-BF, low-energy breakfast condition; %beta, steady state beta cell estimate; %S, insulin sensitivity. ^a^ Body mass was measured on scales while fasted. ^b^ From DXA measures which were only measured at baseline on a single occasion for each participant. ^c^ Fasting values are based on venous blood data from an average of two time points prior to the first meal. ^d^ From SenseWear Armband accelerometer data. ^e^ From activPAL3 monitors during waking hours ≥ 10 h. ^f^ Only measured at baseline as per DXA. No significant differences were observed between pre-condition measures (*p* > 0.05) using students *T*-test.

**Table 2:** Measures of activity calculated from activity monitors worn by individuals with prediabetes (*n* = 13) in response to a high-energy first meal (HE-BF) or a low-energy first meal (LE-BF).

Measure	Time	HE-BF	LE-BF	Difference (95% CI)
Sitting (%) ^a^	Pre-trial	64 ± 11	65 ± 11	−1 (−6, 4)
	Trial day	98 ± 2 *	98 ± 2 *	1 (−4, 6)
	Post-trial	68 ± 10	71 ± 10	−2 (−6, 2)
Standing (%) ^a^	Pre-trial	27 ± 7	26 ± 8	1 (−3, 5)
	Trial day	2 ± 1 *	2 ± 2 *	0 (−5, 4)
	Post-trial	22 ± 8	20 ± 8 ^†^	2 (−2, 6)
Stepping (%) ^a^	Pre-trial	9 ± 6	9 ± 4	0 (−2, 2)
	Trial day	1 ± 1 *	1 ± 1 *	−1 (−3, 1)
	Post-trial	9 ± 5	9 ± 4	0 (−1, 2)
Sitting 30 min blocks (%) ^a^	Pre-trial	46 ± 15	50 ± 15	0 (−2, 2)
	Trial day	96 ± 4 *	98 ± 3 *	−1 (−3, 1)
	Post-trial	57 ± 15	56 ± 23	0 (−2, 2)
Energy expenditure (kJ/day) ^b^	Trial day	9053 ± 1715	8561 ± 1370	613 (−243, 1469)

Data are mean ± SD; from LMM analyses, significantly different (*p* < 0.05) from * pre-trial and post-trial, ^†^ post-trial only. ^a^ From Activpal activity monitors where, Pre-trial: includes 48 h of wear until 08:00 h on trial day; Trial: 08:00 h to 18:00 h inclusive; Post-trial: 18:00 h to 08:00 h following day. ^b^ From SenseWear Armband monitors estimated from total wear time (98%) over 24 h.

**Table 3:** Measures of glycaemia calculated from a continuous blood glucose monitor (CGM). Measurements from individuals with prediabetes (*n* = 13) in response to a high-energy breakfast (HE-BF) or a low-energy breakfast (LE-BF).

Measure	HE-BF	LE-BF	Difference (95% CI)	*p*-Value
AUC_total_ (mmol/h/L)	155.4 ± 15.8	153.5 ± 16.8	1.91 (−7.22, 11.03)	0.66
AUC_total8-6_ (mmol/h/L)	69.8 ± 8.4	65.3 ± 7.4	4.43 (0.64, 8.21)	0.026
Mean glucose (mmol/L)	6.53 ± 0.65	6.45 ± 0.70	0.08 (−0.30, 0.46)	0.66
SD_glucose_ (mmol/L)	0.99 ± 0.34	1.00 ± 0.43	−0.02 (−0.27, 0.24)	0.89
MAGE (mmol/L)	2.48 ± 1.24	2.63 ± 1.27	−0.15 (−1.0, 0.70)	0.71
CONGA-1	6.05 ± 0.62	5.80 ± 0.63	0.25 (0.63, −0.13)	0.17

Data are mean ± SD, analysed using LMM; AUC, area under the curve; AUC_total8-6_, total AUC between 08:00 and 18:00 h for CGM glucose; CONGA-1, continuous overlapping net glycaemic action at 1 h; iAUC, incremental area under the curve; MAGE, mean amplitude glucose excursions; SD, standard deviation.

## Supplementary Materials

The following are available online at http://www.mdpi.com/2072-6643/10/6/733/s1, Table S1: Meal composition and nutritional values for a 10900 kJ (~2600 kcal) example diet for each condition, File S1: CONSORT checklist.
