# Supplementary material for: Impact of First Meal Size during Prolonged Sitting on Postprandial Glycaemia in Individuals with Prediabetes: A Randomised, Crossover Study
Source: Nutrients. 2018 Jun 6;10(6):733. doi: 10.3390/nu10060733 (PMC6024819; doi:10.3390/nu10060733)
Supplement: Supplementary file 1 [file nutrients-10-00733-s001.zip › Parr Prediabetes Nutrients Table S1 160418.pdf]

Table S1. Meal composition and nutritional values for a 10900 kJ example diet for each condition.

	HE-BF							LE-BF						
	Weight (g)	Energy (kJ)	Total CHO (g)	Sugars (g)	Fat* (g)	Protein (g)	Fibre (g)	Weight	Energy (kJ)	Total CHO (g)	Sugars (g)	Fat* (g)	Protein (g)	Fibre (g)
**Breakfast**														
White bread	281	2708	111.9	6.7	5.9	22.5	18.5	113	1083	44.8	2.7	2.4	9.0	7.5
Sliced ham	94	438	1.9	0.8	2.3	15.9	1.7	38	175	0.8	0.3	0.9	6.4	0.7
Butter	9	263	0.0	0.0	7.0	0	0.0	6	188	0	0	5.0	0	0
Cheddar cheese	63	1044	0.1	0.1	21.3	15.0	0.0	25	418	0	0	8.5	6.0	0
Yoghurt	123	478	18.0	18.0	2.0	6.0	0.1	88	341	12.0	12.8	2.0	4.4	0.1
Orange Juice	240	408	21.6	21.6	0	1.4	0.5	150	255	13.5	9.5	0	0.9	0.3
**Breakfast Total**		**4916**	**153.5**	**47.2**	**38.5**	**61.0**	**20.8**		**2277**	**71.1**	**25.3**	**18.8**	**26.7**	**8.6**
**B/fast EI (%)**			**46%**		**27%**	**20%**				**47%**		**28%**	**19%**	
**Lunch**														
English muffins	106	878	37.2	1.8	2.2	7.5	3.3	106	878	37.2	1.8	2.2	7.5	3.3
Baked beans	238	855	30.6	8.4	1.3	11.9	12.1	238	855	30.6	8.4	1.3	11.9	12.1
Butter	9	263	0	0	7.0	0	0	9	263	0.0	0	7.0	0	0
Cheddar cheese	35	585	0	0	11.9	8.4	0	35	585	0	0	11.9	8.4	0
Yoghurt	113	439	15.5	15.5	2.1	5.6	0.1	113	439	15.5	15.5	2.1	5.6	0.1
Tinned fruit (in juice)	156	336	17.2	17.2	0.2	1.2	2.6	156	336	17.2	17.2	0.2	1.2	2.6
**Lunch Total**		**3118**	**100.5**	**42.9**	**24.7**	**34.6**	**18.1**		**3118**	**100.5**	**42.9**	**24.7**	**34.6**	**18.1**
**Lunch EI (%)**			**48%**		**27%**	**18%**				**48%**		**27%**	**18%**	
**Dinner**														
White rice	125	1049	48.8	0.1	3.8	3.8	1.2	288	2412	112.1	0.3	8.6	8.6	2.9
Tuna (in olive oil)	63	576	0	0	8.6	17.4	0	156	1441	0.0	0	21.4	43.4	0
Steamed vegetables	75	92	2.1	2.1	0.2	2.0	2.6	150	183	4.2	4.2	0.3	3.9	5.3
Sweet chili sauce	13	59	2.5	2.0	0.3	0.1	0.6	25	118	4.7	3.8	0.7	0.2	1.1
Butter	5	150	0	0.0	4.0	0	0.0	13	375	0	0.0	10.1	0.1	0.0
Yoghurt	63	244	8.6	8.6	1.2	3.1	0.1	188	731	25.9	25.9	3.6	9.4	0.2
Orange juice	75	128	7	7	0	0	0.2	240	408	21.6	21.6	0	1.4	0.5
**Dinner total**		**2219**	**68.6**	**24.7**	**18.1**	**26.4**	**4.7**		**5487**	**168.5**	**65.2**	**44.6**	**67.1**	**10.0**
**Dinner EI (%)**			**48%**		**29%**	**20%**				**48%**		**29%**	**20%**	
**Daily Total**		**10989**	**321.6**	**114.8**	**81.3**	**121.8**	**43.3**		**11482**	**340.3**	**133.4**	**88.2**	**127.9**	**36.5**

Key: CHO, carbohydrate; HE-BF, high energy breakfast; LE-BF, low energy breakfast. *Fat breakdown for HE-BF: 51%:22%:26%, LE-BF: 58%:15%:27% (Saturated : Monounsaturated : Polyunsaturated; respectively
